# Spherical Trihedral Metallo-Borospherene with Asymmetric Triangles in Boron Framework

**DOI:** 10.3390/nano15221728

**Published:** 2025-11-16

**Authors:** Qin Xie, Weiyi Wang, Qiang Liu, Shufa Li, Lijuan Yan

**Affiliations:** 1 College of Electronics & Information Engineering, Guangdong Ocean University, Zhanjiang 524088, China; xieqin504@gdou.edu.cn (Q.X.); ljyan@gdou.edu.cn (L.Y.); 2 Hefei National Research Center for Physical Sciences at the Microscale, State Key Laboratory of Precision and Intelligent Chemistry, University of Science and Technology of China, Hefei 230026, China; dancingw@ustc.edu.cn

**Keywords:** metallo-borospherenes, asymmetric triangles, stability

## Abstract

The recent discovery of spherical trihedral metallo-borospherenes Ln_3_B_18_^−^ (Ln = La, Tb) represents the onset of an unprecedented class of boron-metal binary nanomaterials, where heterogeneous La or Tb atoms, alongside B atoms, constitute integral components of the outer cage surface. Here, *C_3v_* A_1_-Sc_3_B_16_^+^ is theoretically predicted as the global minimum by the particle swarm optimization (PSO) algorithm, and its B_16_-framework comprises unequal B_6_ and B_7_ triangles, connected by three B atoms between the vertexes of the B_6_ triangle and the interval edges of the B_7_ triangle. This arrangement forms three equivalent η^7^-B_7_ rings, each centered with a Sc atom. The cage surface of the second low-lying A_2_ isomer features an asymmetric triangle of B_3_ and B_7_, along with three B_2_-linked units. This configuration gives rise to three equivalent octacoordinated Sc atoms, each centered within η^8^-B_8_ rings. These two cationic isomers have been proven to be very stable in kinetics and thermodynamics. Our findings regarding unequal boron triangles and smaller odd-coordinated rings in the B-framework enrich the geometric patterns of trihedral metallo-borospherenes.

## 1. Introduction

It is widely acknowledged that carbon’s bonding properties exhibit various forms of sp^3^, sp^2^, and sp bonding, resulting in a wide variety of nanostructures. As the left neighbor of carbon in the periodic table, boron shares similarities in bonding variability and can rival carbon in generating nanostructural analogs. Typical examples include the two-dimensional (2D) graphene [1], stripped from layered graphite, while borophene lacks a large, blocky layered counterpart; experimental synthesis has successfully yielded various structural motifs depending on the substrate [2]. Moreover, corresponding to the structure of bilayer graphene, bilayer borophenes have been obtained experimentally on Cu(111) or Ag(111) substrates, respectively [3,4]. In the case of clusters, the resemblances between them are more obvious. As a counterpart to C_60_ [5], all-boron fullerene *D_2d_* B_40_^−/0^, which is known as borospherenes, has been confirmed experimentally and theoretically [6]. Notably akin to the first axial chiral fullerene *D_2_* C_76_ [7], the first axial chiral borospherene *C_3_*/*C_2_* B_39_^−^ is characterized through photoelectron spectroscopy (PES) measurements [8]. In contrast to the endohedral metallofullerenes M@C_60_ (M = La, Li, Er, Dy, Gd, Ca, Nd, Y) [9,10,11], the endohedral metallo-borospherenes of M@B_40_ (Ca, Sr) and M@B_40_ (M = Sc, Y, La) are theoretically devised [12,13]. Another remarkable property of C_60_ is its ability to encapsulate small molecules, while B_40_^−/0^ has been demonstrated to accommodate H_2_, HF, and H_2_O within its cage [14]. All this indicates the emergence of a new field of scientific research, parallel to that of carbon fullerenes.

With four valence orbitals but only three valence electrons (2s^2^2p^1^), boron is a prototypical electron-deficient element. A common strategy to compensate for this deficiency is to introduce electron-rich heteroatoms into boron-based materials, yielding unique properties and attractive chemical bonds [2,15,16]. This principle is clearly exemplified in free-standing, graphene-like borophene. Although inherently unstable, it can be effectively stabilized by the strategic insertion of elements such as Fe, Cr, Ti, Hf, V, Nb, and Ta [17,18,19,20]. This approach also enables the formation of diverse structural motifs in transition-metal-doped boron clusters, including half-sandwich structures (PrB_7_^−^, CoB_12_^−^, and RhB_12_^−^) [21,22], inverse-sandwich complexes (Ta_2_B_6_^−/0^, Pr_2_B_8_^−^, and La_2_B_n_^−^ (n = 7–9)) [23,24], inverse triple decker La_3_B_14_^−^ [25], boron molecular wheels MB_n_^−^ (CoB_8_^−^, RuB_9_^−^, TaB_10_^−^ and NbB_10_^−^) [26,27], double-ring tubular boron drums MB_n_^−^ (MnB_16_^−^, CoB_16_^−^, RhB_18_^−^, and TaB_20_^−^) [28,29,30,31], and spherical trihedral metallo-borospherenes Ln_3_B_18_^−^ (Ln = La, Tb) [32], etc. Among them, the geometry of *D_3h_* Ln_3_B_18_^−^ stands out as a pioneering discovery in chemistry. This structure features a B_18_ framework comprising two equivalent B_6_ triangles, interconnected by three B_2_ units, forming three conjoined B_10_ rings centered by Ln atoms. Since then, a series of similar derivatives have been rationally designed, including the spherical tetrahedral metallo-borospherene Ta_4_B_18_ [33], the expanded spherical trihedral metallo-borospherenes TM_3_B_15_ (TM = Zr, Hf; q = −1, 0, +1) [34], and Ta_3_B_12_^−^, which exhibits σ + π + δ triple aromaticity [35], etc. These derivatives have a common feature, that is, the B-skeletons are concentrated on B_6_ or B_3_ triangles. Furthermore, a large B_7_ triangle motif has been identified in TM_3/4_B_20_ (TM = Sc, Y) [36]. However, the trihedral metallo-borospherenes structure incorporating other boron motifs remains scarce.

In this work, we successfully designed a novel type of spherical trihedral metallo-borospherenes, namely *C_3v_* A_1_- and A_2_-Sc_3_B_16_^+^ clusters. They both possess an asymmetric B_16_ framework constructed from different regular boron triangles (B_6_/B_7_ or B_3_/B_7_ motifs), along with boron linkers, forming three equivalent η^7^-B_7_ and η^8^-B_8_ rings, respectively. The three Sc atoms were selected as the coordinating centers due to their optimal size match with the boron rings and their shared group with La in the periodic table. These two isomers are located through the particle swarm optimization (PSO) searches. Their high stability is evidenced by the lowest relative energy and substantial energy gaps, along with the geometries retained at 1200K, as demonstrated by molecular dynamics (MD) simulations, etc. Additionally, the simulated Infrared (IR) and Raman spectra are provided to facilitate future experiments.

## 2. Materials and Methods

The entire computational procedure comprises three sequential steps. First, a structural search was conducted using the PSO algorithm as implemented in the CALYPSO 6.0 (Crystal structure AnaLYsis by Particle Swarm Optimization) package [37]. The key parameters configured for this search included: atomic species and their quantities, minimum interatomic distance constraint (DistanceOfIon), population size (PopSize), maximum number of steps (MaxStep), particle swarm ratio (PsoRatio), as well as electron count (Nelect), which determines the charge state of the system. Among them, the PsoRatio parameter governs structural diversity and is typically set to 0.60 to achieve optimal results. The search completed 100 generations, with 30 low-lying isomers identified in each cycle, and ultimately yielded approximately 2500 trial structures for each charged state of Sc_3_B_16_ (cationic, neutral, and anionic), all optimized at the PBE/6-31+G(d) level of theory. Then, the initially obtained low-lying isomers are fully re-optimized using the Gaussian 16 program suite [38], employing combinations of the PBE0 [39] or TPSSh [40] functionals and the basis set of Stuttgart relativistic small-core pseudopotential for Sc atoms and 6-311+G(d) for B atoms, which are usually adopted for boron-based clusters [23,24,32]. Frequency calculations give no imaginary values for all selected isomers, indicating they are true minima on the potential surfaces. The last step involves higher-level energy refinement, where relative energies are recalculated using the more accurate coupled-cluster method with triple excitations, CCSD(T) [41], at PBE0-optimized geometries. In addition, to examine their thermal stability, Born-Oppenheimer molecular dynamics (BOMD) simulations are performed using QUANTUM ESPRESSO 7.3 [42] for 20 ps at the temperatures of 300 K, 1200 K, and 1500 K. MOs and spectra are visualized using Multiwfn_3.8 [43].

## 3. Results

### 3.1. Structures and Stability

**Figure 1:**
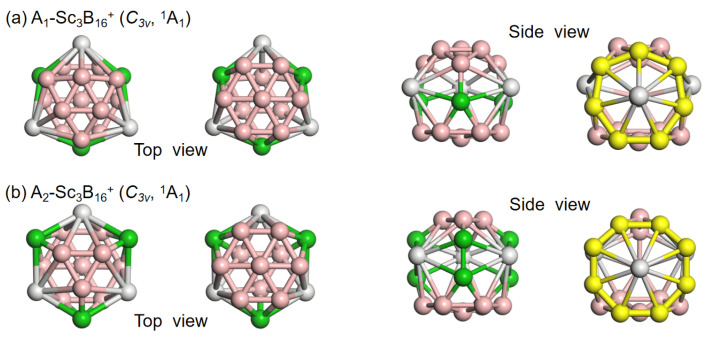
Geometries, symmetries, and electronic states of the two low-lying isomers of Sc_3_B_16_^+^, both of which exhibit a spherical trihedral metallo-borospherene structure. Color code: Sc (white), B (pink), the linking B atoms (green), and the boron rings (yellow).

Considering the boron framework motifs (B_6_, B_3_, and B_7_ triangles) present in the known spherical trihedral metallo-borospherene, we manually construct two types of asymmetric boron frameworks. These are obtained by combining B_6_ and B_7_ triangles, or B_3_ and B_7_ triangles, and the connectors adopt three boron atoms or three B_2_ units, respectively. Aligned with its high stability, a spherical trihedral metallo-borospherene should inherently have an advantage in energy compared to other low-lying isomers. To achieve this objective, we adjust various parameters, including the charges of whole systems, the sizes and configurations of boron skeletons, the types and number of doped heteroatoms, etc. Encouragingly, extensive global minimum (GM) searches of Sc_3_B_16_^+^ successfully yielded two unique structural motifs, both possessing *C_3v_* symmetry, which were assigned as the lowest-lying isomers A_1_ and A_2_ depicted in Figure 1. As expected, A_1_ possesses the B_6_ and B_7_ triangles, as well as the three linked boron atoms form three equivalent conjoined η^7^-B_7_ rings, centered with three Sc atoms. A_2_ isomer has three equivalent conjoined η^8^-B_8_ rings, each with an octacoordinated Sc atom at the center.

**Figure 2:**
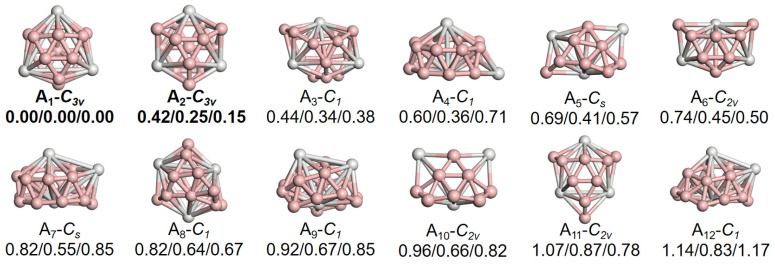
Geometries, symmetries, and relative energies (in eV) of low-lying Sc_3_B_16_^+^ isomers, computed at the PBE0/TPSSh/CCSD(T)//B/6-311+G(d)/Sc/SDD levels. All isomers are labeled (A_1_, A_2_, A_3_, …,) in order of increasing PBE0 relative energy.

The confirmation of these two structures as the most stable isomers is further supported by the relative energies presented in Figure 2, which displays the first twelve low-energy isomers ranked according to their relatively elevated energies at the PBE0 level of calculation. The relative energy of A_2_ is 0.42/0.25/0.15 eV higher than that of A_1_ isomer calculated by PBE0 (left), TPSSh (middle), and CCSD(T) (right) levels, respectively. In the subsequent low-energy isomers, some (e.g., A_3_, A_4,_ and A_5_) lose the equivalent conjoined rings on their cage surfaces, whereas others, notably A_6_ or A_11_ isomers, exhibit relatively high symmetry of *C_2v_*, with the relative energies as high as 0.74/0.45/0.50 eV and 1.07/0.87/0.78 eV, respectively. Such large relative energies indicate markedly reduced stability compared to A_1_ and A_2_. Therefore, none of these isomers corresponds to the structural type we anticipated. Simultaneously, we assess the charge impact on the stability of these two structures, as illustrated in Figure S1i,ii. Regardless of whether they are in a neutral or anionic state, the desired spherical trihedral metallo-borospherene no longer exhibits the lowest energy. It is 0.71/0.88/0.86 and /0.75/1.06/1.00 eV higher in energy than the lowest-lying isomer B_1_ in its neutral state, and 0.68/0.79/0.77 and /1.38/1.58/1.68 eV higher in energy than C_1_ in the anionic state at the calculation of PBE0, TPSSh, and CCSD(T) levels, respectively. Consequently, they also do not possess the high stability characteristic of spherical trihedral metallo-borospherene and thus fall outside the primary concerns of this work.

As we already know, vibrational analysis can be used to evaluate the stability of nanoclusters: real frequencies indicate stability, while imaginary frequencies denote instability. All of the presented structures have real frequencies, suggesting their high stability and realizability. To judge the stability of small molecules, Hoffmann et al. have proposed more accurate criteria, in which the true value of the smallest vibrational frequency should be reasonably large, 100 cm^−1^ or more [44]. The lowest vibrational frequency of A_1_ and A_2_ isomers is 246 or 248 cm^−1^, respectively, which are sufficient to meet the stability criterion and indicate their good dynamical stability. The neutral counterparts exhibit frequencies of 90 and 93 cm^−1^, while the anionic counterparts display frequencies of 121 and 166 cm^−1^, respectively, significantly smaller than those of A_1_ and A_2_, indicating their lower stability compared to the corresponding cationic states. Furthermore, the energy gap (E_gap_), defined as the energy difference between the highest occupied molecular orbital (HOMO) and the lowest unoccupied molecular orbital (LUMO), i.e., E(LUMO) − E(HOMO), is another useful measure for assessing the kinetic stability of small clusters. A significant energy gap correlates with a heightened energy demand for electron excitation. The calculated E_gap_ of both are 2.33 and 2.55 eV, respectively, potentially indicating high stability. As for their neutral and anionic counterparts with spherical trihedral metallo-borospherene structures, the gaps are smaller, where the neutral isomers B_3_ and B_4_ have gaps of 1.60 and 1.55 eV, and the anionic isomers C_5_ or C_6_ have gaps of 1.68 and 1.36 eV at PBE0 levels, respectively, all suggesting lower stability.

**Figure 3:**
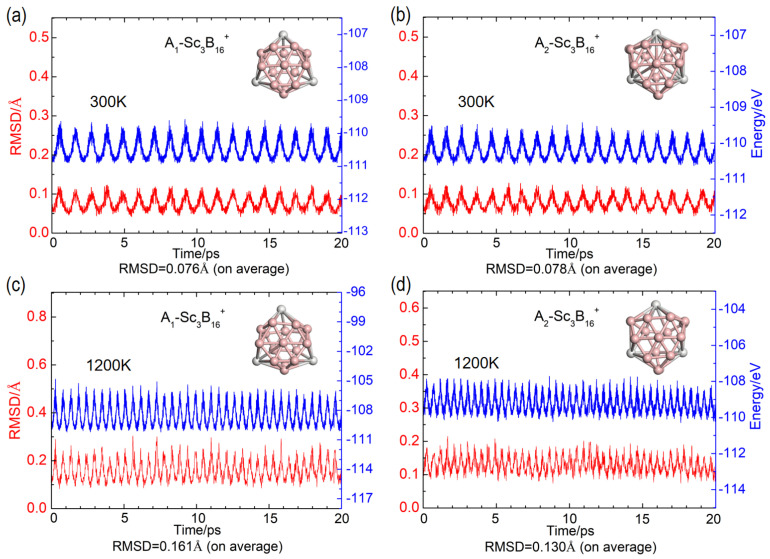
BOMD trajectory and RMSD of *C_3v_* Sc_3_B_16_^+^ obtained for a time of 20 ps at 300 K for (**a**) A_1_-Sc_3_B_16_^+^, RMSD = 0.076 Å (on average); (**b**) A_2_-Sc_3_B_16_^+^, RMSD = 0.078 Å (on average); and at 1200 K for (**c**) A_1_-Sc_3_B_16_^+^, RMSD = 0.161 Å (on average); (**d**) A_2_-Sc_3_B_16_^+^, RMSD = 0.130 Å (on average).

We additionally investigate the thermal stabilities of A_1_ and A_2_ isomers by BOMD, a method allowing for the analysis of stability through tracking changes in total energy, motion trajectory, and structure across various temperatures over a period of time. At the temperature of 300 K, the two lowest-lying isomers retain their integrity, with no collapse in geometry observed. Specifically, over a period of 20 ps with a time step of 1 fs, there is no significant distortion in the positions of three Sc atoms and the shapes of B_16_-frameworks. As shown in Figure 3a,b, the total potential energies for these two cage-like isomers exhibit minimal variation, approximately 0.05 eV/atom (blue lines), a slight fluctuation around a certain constant magnitude. At the same time, the average root mean square deviations (RMSD) are also small, with 0.076 Å for the A_1_ isomer and 0.078 Å for the A_2_ isomer (red lines). Even at 1200 K, the basic structural patterns of both isomers can be maintained, with RMSD of 0.161 and 0.130 Å, respectively. When the two isomers are in neutral or anionic states, their RMSD shows significant distortions and they even transform into another geometry as depicted in Figure S2. Actually, at a higher temperature of 1500 K, the geometry of cationic A_1_ isomer can still be retained with an RMSD of 0.171 Å. Conversely, the cationic A_2_ isomer experiences significant variation with an RMSD of 1.021 Å, swiftly transitioning to another geometry as shown in Figure S3. This result underscores the superior stability of the cationic A_1_ isomer over A_2_, which is fully consistent with the computed relative energies. Specifically, the A_1_ isomer is more stable than A_2_ by 0.42 eV (PBE0), 0.25 eV (TPSSh), and 0.15 eV (CCSD(T)), respectively. These findings collectively establish A_1_ as a more promising candidate for synthesis than the A_2_ isomer.

### 3.2. Bonding Pattern Analyses

Obviously, the *C_3v_* A_1_- and A_2_-Sc_3_B_16_^+^ isomers, featuring spherical trihedral metallo-borospherene structures, exhibit higher stability than the other low-energy isomers, as determined by the above energy calculations and stability analysis. Regardless of its charge state (neutral, anionic, or cationic), the most stable isomer of B_16_ is invariably either a quasi-planar structure or a double ring structure that competes with a planar structure [15,45,46]. To elucidate the origin of the high stability in these two configurations, their bonding natures are analyzed. Generally, the B–B bonds on the periphery are localized two-center–two-electron (2c–2e) σ bonds, while the delocalized multi-center–two-electron (nc–2e) σ and π bonds occur within the interior or throughout the entire skeleton.

**Figure 4:**
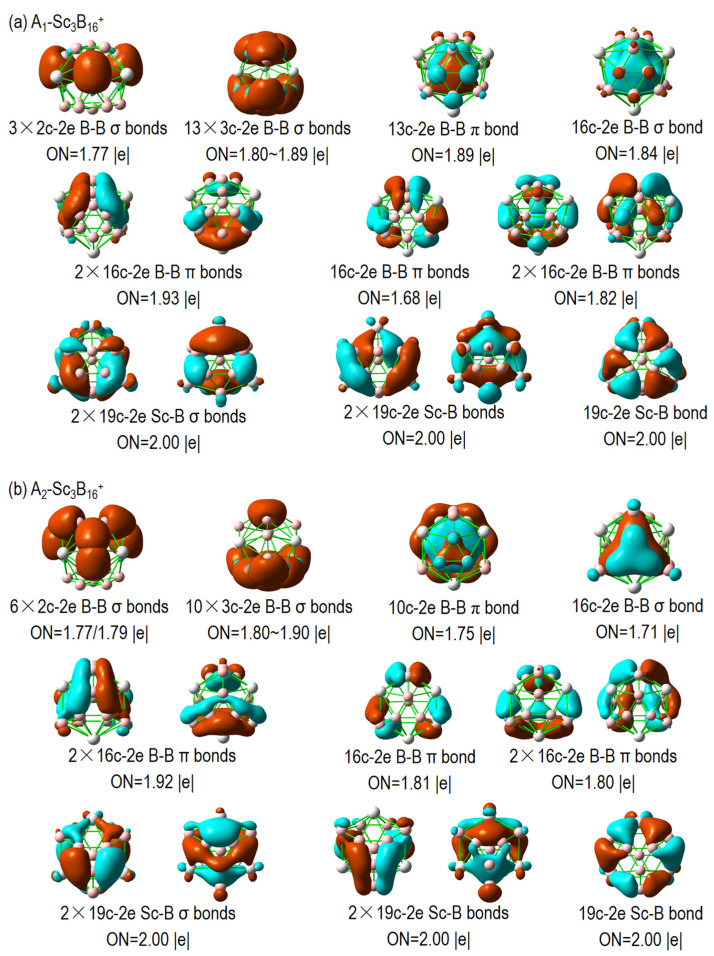
AdNDP bonding patterns of *C_3v_* (**a**) A_1_-Sc_3_B_16_^+^ and (**b**) A_2_-Sc_3_B_16_^+^ at PBE0 level.

As shown in Figure 4, the *C_3v_* A_1_ isomer has three 2c–2e B–B σ bonds, connecting the three linked B atoms with the three apexes of the B_6_ triangle, each with an occupation number (ON) of 1.77 |e|. The σ–skeleton of the cage-like surface also includes thirteen delocalized 3c–2e B–B σ bonds, of which four reside in the B_6_ triangle, six in the B_7_ triangle, and three between the linked B atoms and the B_7_ triangle. The ON falls within the range of 1.80~1.89 |e|. The multi-center 13c–2e and 16c–2e B–B delocalized bonds, which span the B_6_ and the B_7_ units, can be viewed as π or σ bonds, respectively. The subsequent bonds in the second row represent totally delocalized π bonds and σ bonds of the B_16_ framework. The remaining five totally delocalized 19c–2e Sc–B bonds exist between the three Sc atoms and the B_16_ framework. Despite the different geometry with the A_1_ isomer, the bonding pattern of the *C_3v_* A_2_ isomer is nearly identical to that of the A_1_ isomer, with the main discrepancy being the distributions of skeleton bonds and ONs: specifically, three 2c–2e and thirteen 3c–2e B–B σ for A_1_, versus six 2c–2e and ten 3c–2e B–B σ for A_2_. The significant differences in ON values are primarily due to delocalized bonds within the boron framework, arising from variations in geometric patterns in B_16_.

### 3.3. Aromaticity

**Figure 5:**
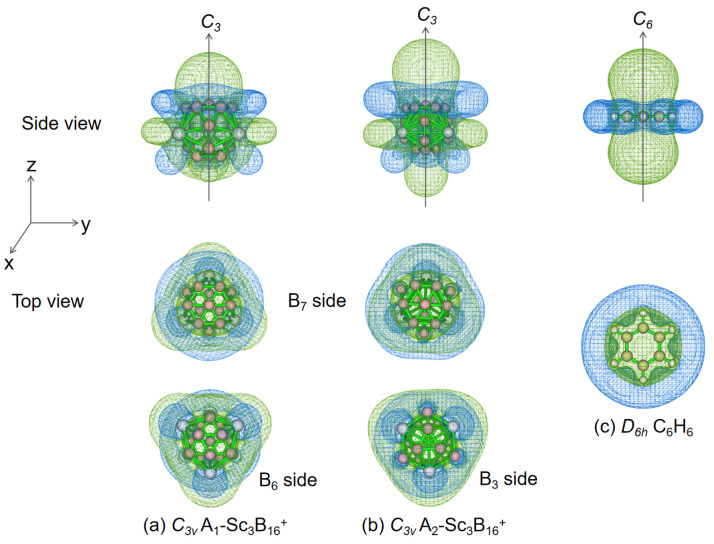
Calculated ICSSs (isovalue: ±2 ppm) of *C_3v_* Sc_3_B_16_^+^ with (**a**) A_1_ and (**b**) A_2_ geometric structures, and (**c**) *D_6h_* C_6_H_6_, all of which have the corresponding NICS-ZZ components indicated. The *C_3_* axis of A_1_ and A_2_ isomers around their two triangles of B_7_ and B_6_, or B_7_ and B_3_, and the *C_6_* axis of C_6_H_6_ is designated as the z axis in the vertical direction. The chemical shielding and de-shielding areas are indicated in green or blue, respectively.

For spherical clusters, the nucleus-independent chemical shift (NICS) is often used to assess aromaticity, a concept first proposed by von Schleyer in 1966 to characterize the aromaticity of organic compounds [47,48]. It can be obtained by introducing a virtual atom (ghost atom) B_q_ at an arbitrarily selected location, where negative NICS indicates aromaticity and positive NICS indicates antiaromaticity. To vividly demonstrate chemical shielding, we have computed the magnetic shielding tensor at evenly distributed grid points surrounding the system and generated specific tensor components as iso-chemical shielding surfaces (ICSSs) [49,50]. Based on the calculated NICS-ZZ components, a three-dimensional isosurface map is plotted to demonstrate the chemical shielding shown in Figure 5, with the z direction aligned parallel to the *C_3_* molecular axis. The chemical shielding area of A_1_-Sc_3_B_16_^+^ is divided into two parts by the Sc_3_ plane (Figure 5a). At the side of the B_7_ triangle, the chemical shielding area with negative NICS-ZZ values extends from the interior to a vertical interval of about 1.0 Å above the B_7_ triangle, highlighted in green, while the chemical de-shielding regions with positive NICS-ZZ values are horizontally surrounding around the B_7_ triangle, with the color of blue and the shape of a belt-like. At the B_6_ triangle side, the chemical de-shielding region is intermittently distributed, failing to fully enclose the B_6_ triangle horizontally, indicating relatively weaker local aromaticity. In brief, the most stable isomer of Sc_3_B_16_^+^ is partially aromatic. The second low-lying isomer A_2_-Sc_3_B_16_^+^, which is with the combination of B_3_ and B_7_ triangles on the cage surface, also possesses a local aromaticity as shown in Figure 5b. Despite the disparity in geometries, A_1_- and A_2_-Sc_3_B_16_^+^ exhibit similar aromaticity, probably because both of them have a nearly circular B_7_ triangle. From the top view, their ICSSs’ behaviors closely resemble that of typical aromatic benzene C_6_H_6_ (Figure 5c), confirming the aromatic character. It is worth noting that the side view reveals a distinct separation of the blue-marked chemical de-shielding region, clearly different from benzene and other spherical trihedral metallo-borospherenes, such as *D_3h_* Ta_3_B_12_^−^ [35], *T_d_* Ta_4_B_18_ [33], *D_3h_* La@[La_5_&B_30_] [51], etc. The possible cause is asymmetric triangles within the boron skeleton. Hence, the isoelectronic *D_3h_* Sc_3_B_15_^2−^ with two symmetrical triangular B_6_ triangles is calculated, and the chemical deshielding region shows an intermittent distribution, yet from the side it appears as a continuous band (Figure S4).

### 3.4. Simulated IR and Raman Spectra

**Figure 6:**
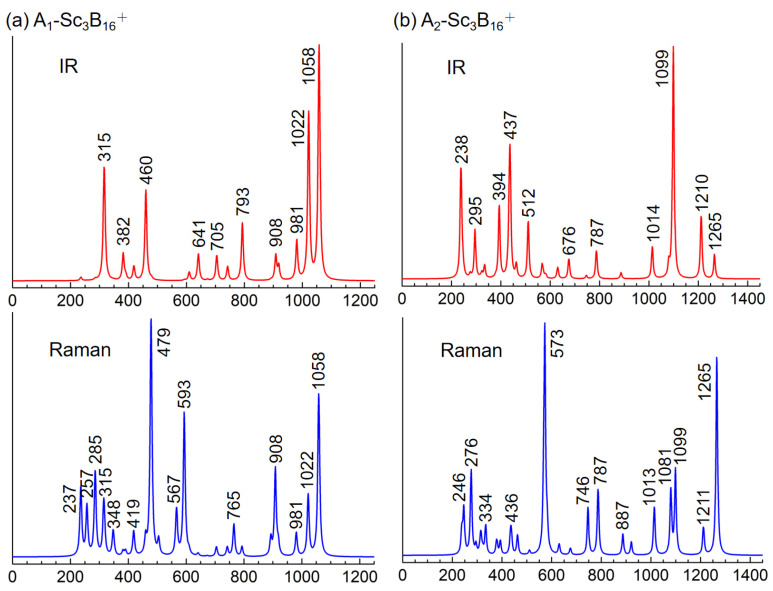
Simulated IR and Raman spectra of *C_3v_* (**a**) A_1_-Sc_3_B_16_^+^ and (**b**) A_2_-Sc_3_B_16_^+^ at PBE0/6-311+G(d) level (units: cm^−1^; scaling factors of 0.958).

To facilitate the experimental characterizations in the future, the IR and Raman spectra of *C_3v_* A_1_- and A_2_-Sc_3_B_16_^+^ are computationally simulated at the PBE0/6-311+G(d) level. Since the calculated harmonic frequencies systematically overestimate experimental values, a standard scaling procedure (ν_scaled_ = Scaling Factor × ν_calculated_) was applied [52,53]. The spectra in Figure 6 were consequently generated by scaling the raw frequencies with a factor of 0.958, as recommended by the authoritative NIST Computational Chemistry Comparison and Benchmark Database (CCCBDB) [54]. As shown, the lowest-lying isomer exhibits five major asymmetrical IR vibrations at 315, 460, 793, 1022, and 1058 cm^−1^, and five symmetrical Raman vibrations at 479, 593, 908, 1022, 1058 cm^−1^, as well as numerous relatively weak Raman spectral features within the range of 237~419 cm^−1^. Detailed vibrational analyses reveal that the two symmetrical vibrations at 257 and 593 cm^−1^ represent typical radial breathing modes (RBMs) of three Sc atoms and the B_16_-framework on the cage-like surface, respectively. Such RBM spectral features are distinctive, much as they are in characterizing single-walled hollow boron nanostructures [55]. As for the A_2_ isomer, the IR peaks mainly occur at 238, 295, 394, 437, 512, 1099, and 1210 cm^−1^. The Raman-active vibrations include two major peaks at 573 and 1265 cm^−1^, along with a series of minor vibrations at 246, 276, 746, 787, 1013, 1081, and 1099 cm^−1^, etc. Among them, the two Raman vibrations at 246 and 573 cm^−1^ are identified as its RBMs. Despite both A_1_- and A_2_-Sc_3_B_16_^+^ having *C_3v_* cage-like structures, geometric differences significantly influence their spectral characteristics and allow for distinction through these analytical methods.

## 4. Conclusions

In this work, we design a new type of spherical trihedral metallo-borospherene A_1_-and A_2_-Sc_3_B_16_^+^, which is typically characterized by the combinations of asymmetric B_6_ and B_7_ triangles, or B_3_ and B_7_ triangles, as well as boron units, in the outer B_16_-framework. The two envisaged geometries were identified as the lowest-lying isomers by the PSO algorithm, as well as by PBE0, TPSSh, and CCSD(T) calculations, which consistently show that these structures possess the lowest relative energies. Their high stability is confirmed by the facts of real frequencies, especially the minimum vibrational frequencies with large values of 246 and 248 cm^−1^, and the geometries maintained at 1200 K. By adjusting the boron framework with asymmetric triangles, it is possible to introduce novel electronic, magnetic, and optical properties to spherical trihedral metallo-borospherene nanoclusters, thereby extending their applications.

## Data Availability

The data presented in this study are available on request from the corresponding author.

## Supplementary Materials

The following supporting information can be downloaded at: https://www.mdpi.com/article/10.3390/nano15221728/s1. Figure S1: Geometries, symmetries and relative energies of the low-lying isomers of neutral and anionic Sc_3_B_16_; Figure S2: RMSD of *C_3v_* Sc_3_B_16_ in neutral (a,b) and anionic (c,d) states obtained for a time of 20 ps at the temperatures of 1200 K; Figure S3: BOMD trajectory and RMSD of *C_3v_* A_1_- and A_2_-Sc_3_B_16_^+^ obtained for a time of 20 ps at the temperatures of 1500 K; Figure S4: Calculated ICSSs of (a) *C_3v_* Sc3B_15_^2−^ and (b) *D_6h_* C_6_H_6_; Table S1: Cartesian coordinates of the first six low-lying isomers of Sc_3_B_16_^+^ at PBE0/6-311+G(d) level.
