# Supplementary material for: Spherical Trihedral Metallo-Borospherene with Asymmetric Triangles in Boron Framework
Source: Nanomaterials (Basel). 2025 Nov 16;15(22):1728. doi: 10.3390/nano15221728 (PMC12655609; doi:10.3390/nano15221728)
Supplement: Supplementary file 1 [file nanomaterials-15-01728-s001.zip › nanomaterials-3953217-supplementary.pdf]

Supplementary Information

Spherical Trihedral Metallo-Borospherene with Asymmetric Triangles in Boron Framework

Qin Xie [1], Weiyi Wang [2], Qiang Liu [1,*], Shufa Li [1,*] and Lijuan Yan [1]

[1] College of Electronics & Information Engineering, Guangdong Ocean University, Zhanjiang 524088, China; xieqin504@gdou.edu.cn (Q.X.); ljyan@gdou.edu.cn (L.Y.)

[2] Hefei National Research Center for Physical Sciences at the Microscale, State Key Laboratory of Precision and Intelligent Chemistry, University of Science and Technology of China, Hefei 230026, China; dancingw@ustc.edu.cn

* Correspondence: liuqiang@gdou.edu.cn (Q.L.); lishufa@gdou.edu.cn (S.L.)

Figure S1 Geometries, symmetries and relative energies of the low-lying isomers of neutral and anionic Sc_3B_{16}.

Figure S2 RMSD of C_{3v} Sc_3B_{16} in neutral (a, b) and anionic (c, d)states obtained for a time of 20 ps at the temperatures of 1200 K.

Figure S3 BOMD trajectory and RMSD of C_{3v} A_1- and A_2-$Sc_3B_{16}^+$ obtained for a time of 20 ps at the temperatures of 1500 K.

Figure S4 Calculated ICSSs of (a) C_{3v} $Sc_3B_{15}^{2-}$ and (b) D_{6h} C_6H_6.

Table S1 Cartesian coordinates of the first six low-lying isomers of $Sc_3B_{16}^+$ at PBE0/6-311+G(d) level.

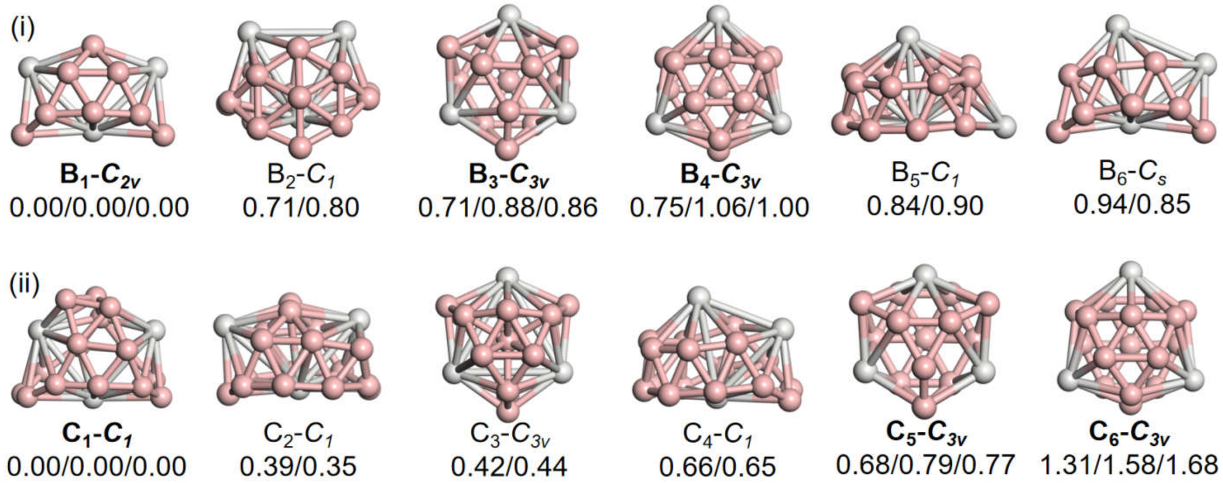

Figure S1 Optimized geometries and symmetries of low-lying (i) neutral and (ii) anionic Sc3B16 clusters. Relative energies (in eV) obtained at PBE0/TPSSh/CCSD(T)//B/6-311+G(d)/Sc/SDD levels are provided. The isomers are labeled in order of increasing PBE0 energy as B_1, B_2, B_3, ..., for neutrals and C_1, C_2, C_3, ... for anions. Notably, the considerable energy gaps between the spherical trihedral metallo-borospherene structures (B_3, B_4, C_5, C_6) and the global minima (B_1, C_1) indicate that the former are unlikely to be the most stable isomers. Consequently, single-point CCSD(T) calculations were conducted on the most relevant isomers to balance computational cost and achieving high accuracy.

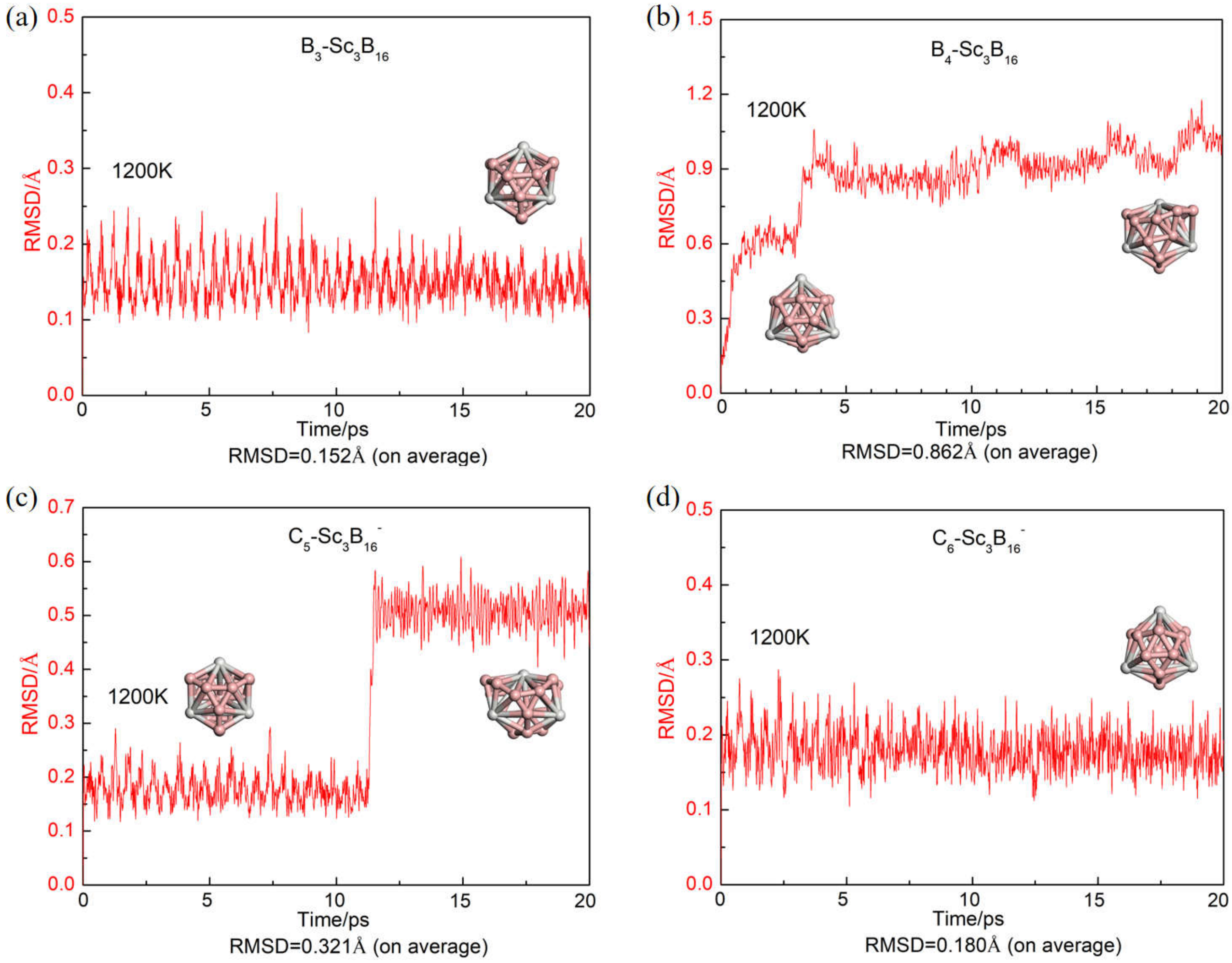

Figure S2 RMSD of C_{3v} Sc_3B_{16} in neutral (a, b) and anionic (c, d) states obtained for a time of 20 ps at the temperatures of 1200 K. Neutral (a) B_3-Sc_3B_{16}, RMSD=0.152 Å (on average) and (b) B_4-Sc_3B_{16}, RMSD=0.862 Å (on average); anionic (c) C_5-$Sc_3B_{16}^-$, RMSD=0.321 Å (on average) and (d) C_6-$Sc_3B_{16}^-$, RMSD=0.180 Å (on average).

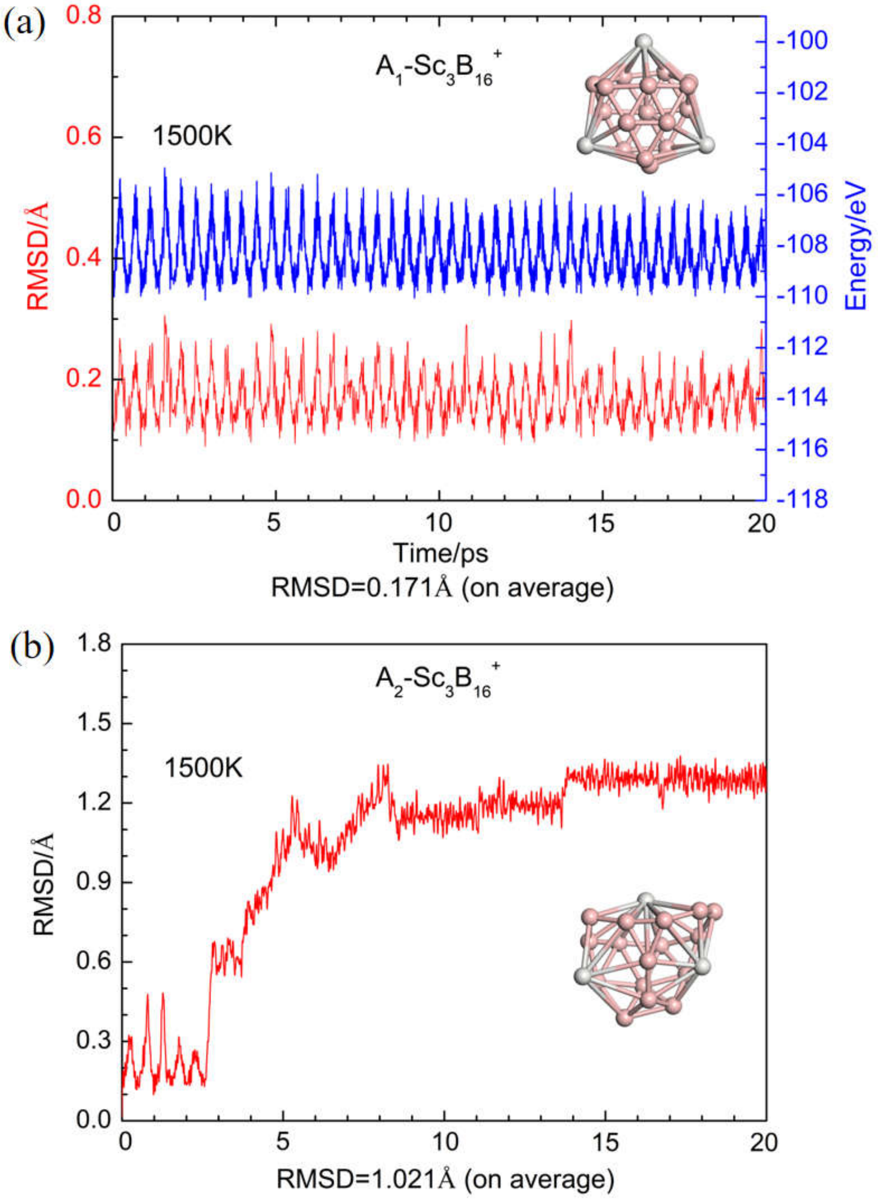

Figure S3 BOMD trajectory and RMSD of C_{3v} $Sc_3B_{16}^+$ obtained for a time of 20 ps at the temperatures of 1500 K for (a) A_1-$Sc_3B_{16}^+$, RMSD=0.171 Å (on average); (b) A_2-$Sc_3B_{16}^+$, RMSD=1.021 Å (on average).

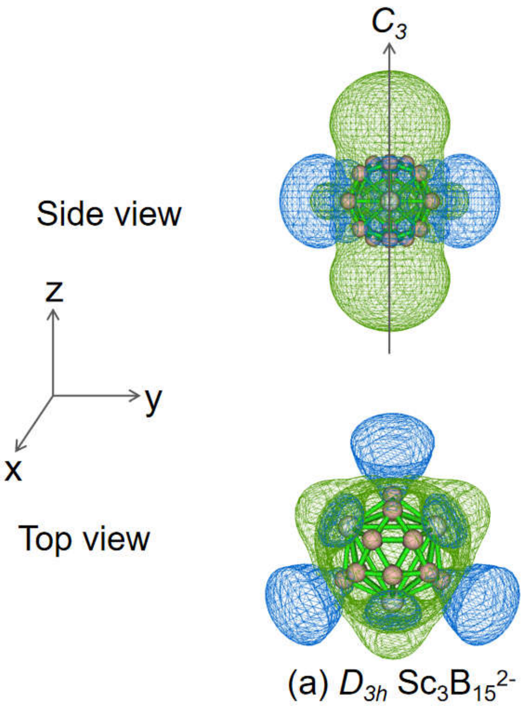

Figure S4 Calculated ICSSs (isovalue: ±2 ppm) of (a) C_{3v} $Sc_3B_{15}^{2-}$ and (b) D_{6h} C_6H_6, all of which the corresponding NICS-ZZ components are indicated. The C_3 axis of $Sc_3B_{15}^{2-}$ around the B_6 triangles and C_6 axis of C_6H_6 are designated as the z axis in vertical direction. The chemical shielding and de-shielding areas are indicated in green or blue, respectively.

Cartesian coordinates of the first six low-lying isomers of $Sc_3B_{16}^+$ at PBE0/6-311+G(d) level.

A$_1$-*C$_{3v}$*

Sc	-0.21087100	-2.43902600	-0.26407800
Sc	-2.00876600	1.40160600	-0.25464600
Sc	2.21713500	1.03830200	-0.27025800
B	1.45079400	-1.01539700	-1.27701600
B	-1.61252300	-0.75154500	-1.26493300
B	0.14737000	1.76986800	-1.27005200
B	0.00670800	-0.00142000	1.87580600
B	0.97462000	1.37022900	1.64271600
B	0.70957200	-1.52606300	1.64103700
B	-1.66633500	0.15196300	1.65101200
B	-0.09147000	-0.97997500	-1.88473000
B	-0.81450500	0.56500500	-1.88078100
B	0.88470100	0.41860900	-1.88608700
B	-1.88713600	-0.88396900	0.35053100
B	0.17973400	2.07670100	0.34486200
B	1.71112100	-1.19411200	0.33637100
B	-0.94806600	-1.38311300	1.64725800
B	-0.71390400	1.51609500	1.64913900
B	1.67982700	-0.13658200	1.63858900

A$_2$-*C$_{3v}$*

Sc	-0.38776300	2.21159900	0.00000000
Sc	-0.38537900	-1.10658100	1.91635600
Sc	-0.38537900	-1.10658100	-1.91635600
B	1.96960900	0.00206800	0.00000000
B	1.70143800	-0.01599600	1.67141600
B	-1.89925100	0.49146100	0.85454700
B	0.52783700	-2.25466100	0.00000000
B	0.52607500	1.12853000	-1.95471600
B	1.70143800	-0.01599600	-1.67141600
B	1.70143800	-1.43647400	-0.85082700
B	-1.06356800	1.13138900	1.96231000
B	0.52607500	1.12853000	1.95471600
B	-1.06184500	-2.26601100	0.00000000
B	-1.90047000	-0.98812100	0.00000000
B	1.69919500	1.45774100	-0.82023600
B	1.69919500	1.45774100	0.82023600
B	-1.89925100	0.49146100	-0.85454700
B	-1.06356800	1.13138900	-1.96231000
B	1.70143800	-1.43647400	0.85082700

A$_3$-*C$_1$*

Sc	1.82492900	-1.30830800	-0.57056700
Sc	0.54201600	1.73513300	0.00371700
Sc	-2.55120600	-0.43639600	-0.66180900
B	-0.35090300	-0.62001700	1.93321200
B	0.80807000	0.32881500	-1.99878500
B	-1.44011400	-1.58004600	1.01402800
B	1.31243900	-0.93635400	1.68023200
B	-0.73403700	1.05020200	2.01234000
B	-0.30336700	-0.78051900	-1.64607700
B	2.22700400	0.83237400	-1.51018900
B	-0.81284600	0.79700200	-1.73164200
B	0.83631600	0.60415800	2.16755600
B	-1.89421200	-0.08630900	1.52798300
B	0.20752000	-1.97563600	1.01718000
B	-0.58760500	-1.81307500	-0.37354700
B	2.82313100	0.85224100	-0.13025000
B	-1.74838800	1.64408600	-0.80848700
B	2.19843400	0.35062700	1.23746600
B	-1.76754400	1.37264900	0.76934700

A$_4$-*C$_1$*

Sc	-0.05302700	0.33164700	-1.03493400
Sc	-0.89967700	-0.59235200	1.80091700
Sc	2.97914300	0.02387400	-0.32524500
B	0.19168200	1.40598800	1.37322800
B	-2.42192200	0.51177200	0.31270700
B	-1.29940200	-2.15060600	-0.12434600
B	-1.29227600	1.63260100	0.68376700
B	1.58216400	1.67383700	0.42333200
B	-1.66750200	-1.36785500	-1.59869200
B	1.39860100	-1.58067400	-0.84039500
B	-2.13892100	1.63300000	-1.00467000
B	-0.16412100	-2.01966700	-1.36239700
B	0.24025800	2.57264100	0.10601000
B	-2.44813900	0.08075400	-1.36715200
B	1.35870200	-1.19975900	0.80166300
B	-2.39904500	-1.01553500	-0.10091100
B	1.36308300	0.30708100	1.31294100
B	-1.05407500	2.70702900	-0.73108400
B	0.23987200	-2.19591800	0.26489900

A_5-C_s

Sc	-0.35048100	-0.00008800	-1.30888300
Sc	-1.89040300	0.00005800	1.32506700
Sc	2.53364100	0.00006200	0.95952000
B	-0.18931000	-2.42693400	-0.74868600
B	0.41957800	-0.80099800	1.51948100
B	-1.73362600	1.88278200	-0.30742200
B	1.14480200	2.07114800	-1.50308800
B	-0.18934500	2.42686500	-0.74890200
B	-0.60319200	1.86782600	0.81641400
B	1.04243700	1.61490200	0.14616900
B	1.04246200	-1.61493400	0.14634300
B	-0.60319600	-1.86776200	0.81655700
B	2.00791800	0.83081600	-1.14432700
B	-2.70348700	0.75435100	-0.82780600
B	-2.70348100	-0.75441300	-0.82775500
B	2.00800300	-0.83092500	-1.14426000
B	1.14484800	-2.07126000	-1.50289100
B	-1.73363400	-1.88281200	-0.30728000
B	0.41964100	0.80121000	1.51949600

A_6-C_{2v}

Sc	-0.00012600	1.36449000	0.00000000
Sc	0.00004800	-0.95824900	2.20167000
Sc	0.00004800	-0.95824900	-2.20167000
B	-2.27500000	0.55886400	0.00000000
B	-0.80019200	-1.72885900	0.00000000
B	1.83445400	0.61765200	1.61569800
B	1.83445400	0.61765200	-1.61569800
B	2.27490600	0.55906200	0.00000000
B	1.88578700	-0.82270600	0.87769800
B	1.88578700	-0.82270600	-0.87769800
B	-1.88570900	-0.82284800	-0.87769400
B	-1.88570900	-0.82284800	0.87769400
B	0.76104100	1.36976500	-2.45943400
B	0.76104100	1.36976500	2.45943400
B	-0.76107200	1.36970300	2.45942800
B	-0.76107200	1.36970300	-2.45942800
B	-1.83446500	0.61751500	-1.61569000
B	-1.83446500	0.61751500	1.61569000
B	0.80033600	-1.72879000	0.00000000
